# Parthenogenetic activation of bovine oocytes using bovine and murine phospholipase C zeta

**DOI:** 10.1186/1471-213X-8-16

**Published:** 2008-02-19

**Authors:** Pablo J Ross, Zeki Beyhan, Amy E Iager, Sook-Young Yoon, Christopher Malcuit, Karl Schellander, Rafael A Fissore, Jose B Cibelli

**Affiliations:** 1 Cellular Reprogramming Laboratory, Department of Animal Science, Michigan State University, East Lansing, MI 48824, USA; 2 Veterinary and Animal Science, University of Massachusetts, Amherst, MA 01003, USA; 3 Institute of Animal Science - Animal Breeding and Husbandry Group, Faculty of Agriculture, University Bonn, Bonn 53115, Germany; 4 Department of Physiology, Michigan State University, East Lansing, MI 48824, USA; 5 Programa Andaluz de Terapia Celular, Andalucia, Spain

## Abstract

**Background:**

During natural fertilization, sperm fusion with the oocyte induces long lasting intracellular calcium oscillations which in turn are responsible for oocyte activation. PLCZ1 has been identified as the factor that the sperm delivers into the egg to induce such a response. We tested the hypothesis that PLCZ1 cRNA injection can be used to activate bovine oocytes.

**Results:**

Mouse and bovine PLCZ1 cRNAs were injected into matured bovine oocytes at different concentrations. Within the concentrations tested, mouse PLCZ1 injection activated bovine oocytes at a maximum rate when the pipette concentration of cRNA ranged from 0.25 to 1 μg/μL, while bovine PLCZ1 was optimal at 0.1 μg/μL. At their most effective concentrations, PLCZ1 induced parthenogenetic development at rates similar to those observed using other activation stimuli such as Ionomycin/CHX and Ionomycin/DMAP. Injection of mouse and bovine PLCZ1 cRNA induced dose-dependent sperm-like calcium oscillations whose frequency increased over time. Injection of bovine and mouse PLCZ1 cRNA also induced IP_3_R-1 degradation, although bovine PLCZ1 cRNA evoked greater receptor degradation than its mouse counterpart.

**Conclusion:**

Injection of PLCZ1 cRNA efficiently activated bovine oocytes by inducing a sperm-like calcium oscillatory pattern. Importantly, the high rate of aneuploidy encountered in parthenogenetic embryos activated by certain chemical means was not observed in PLCZ1 activated embryos.

## Background

Ovulated mammalian oocytes are arrested at the metaphase II (MII) stage of meiosis and only complete meiosis after fertilization. The sperm is responsible for releasing the oocyte from its meiotic arrest, and also for inducing other events that are collectively referred to as oocyte activation. Oocyte activation events include cortical granule exocytosis, reinitiation of meiosis, extrusion of the second polar body, formation of pronuclei, and recruitment of mRNA [1,2]. In all mammalian species studied so far, oocyte activation is triggered by repetitive rises in the intracellular concentration of free Ca^2+ ^([Ca^2+^]_i_) [3], a sufficient and indispensable event [4]. The [Ca^2+^]_i _rises are generated by release of Ca^2+ ^from the intracellular stores, which is mediated by production of inositol 1,4,5-triphosphate (IP_3_) following activation of the phosphoinositide signaling pathway [5,6].

It is hypothesized that upon fusion with the oocyte the sperm introduces a protein factor responsible for inducing production of IP_3 _and Ca^2+ ^release. A growing body of evidence suggests that the sperm factor is phospholipase C-zeta (PLCZ1) [7]. This PLC variant is sperm specific [8] and induces sperm-like [Ca^2+^]_i _oscillations when injected into mouse oocytes [9]. Injection of cRNA coding for PLCZ1 into mature mouse [8], human [10], and pig [11] oocytes induces [Ca^2+^]_i _oscillations and oocyte activation. In mouse sperm, PLCZ1 localizes to the postacrosomal region [9], the area thought to first interact with the oocyte membrane [12]. Functional studies using RNAi to reduce the level of PLCZ1 in sperm showed that [Ca^2+^]_i _oscillations were reduced after intracytoplasmic sperm injection (ICSI) and a lower number of progeny was obtained after natural mating [13]. Finally, in fractionation studies, the presence of immunoreactive PLCZ1 correlated with the ability of fractions to induce oocyte activation [9], and immunodepletion of PLCZ1 from sperm extracts suppressed its [Ca^2+^]_i _oscillation-inducing ability [8]. Altogether, this evidence suggests that PLCZ1 is the factor responsible for oocyte activation in mammals.

PLCZ1, like other PLCs, catalyzes the hydrolysis of phosphatidyl 4,5-bisphosphate (PIP_2_), producing IP_3 _and 1,2-diacylglycerol (DAG). The elevation in IP_3 _concentration is responsible for inducing Ca^2+ ^release from the endoplasmic reticulum (ER), the Ca^2+ ^store of the cell, upon binding its cognate receptor, IP_3_R-1, which is mostly located in this organelle. Continuous production of IP_3 _is thought to underlie the persistence of the oscillations during mammalian fertilization [7,14,15], and eventually lead to IP_3_R-1 degradation [16,17]. IP_3_R-1 downregulation, which is a hallmark of fertilization, is thought to contribute to the decreased responsiveness to IP_3 _observed after fertilization [18]. Importantly, while PLCZ1 has been shown to trigger [Ca^2+^]_i _oscillations, whether or not it is capable of inducing IP_3_R-1 degradation has not been previously reported.

Parthenogenesis is the development of an embryo without paternal contribution [19]. When placed in the uterus of a surrogate mother, mammalian parthenogenetic embryos will develop to different stages depending on the species, but never to term [20]. Bovine oocytes can be parthenogenetically activated using ionomycin, ionophore, ethanol, or electric stimuli [21]. All of these compounds will trigger a monotonic [Ca^2+^]_i _increase that, while necessary, is not sufficient to completely downregulate the activity of Maturation-Promoting Factor (MPF). To accomplish this goal, these [Ca^2+^]_i _releasing agents must be used in combination with a protein synthesis or protein kinase inhibitor such as cycloheximide or 6-dimethylaminopurine (DMAP), respectively [21]. Using these activation protocols, parthenotes can reach the blastocyst stage at reasonable rates; however, the impact these treatments have on *in vivo *development has not been studied, mainly because parthenogenetic embryos are inherently limited in their developmental capacity.

In cattle, PLCZ1 is detected at the equatorial region of sperm [22], and injection of mouse PLCZ1 cRNA induced [Ca^2+^]_i _oscillations in bovine oocytes [23]. Nevertheless, the potential of bovine PLCZ1 cRNA to induce [Ca^2+^]_i _oscillations and parthenogenetic activation in homologous oocytes has not been reported.

In this study, we examined the calcium oscillation-inducing activity of mouse and bovine PLCZ1 cRNA when injected into bovine oocytes, as well as the effect of their injection on IP_3_R-1 concentration. We also compared the developmental rates of oocytes activated using mouse and bovine PLCZ1 cRNA to those observed following activation with commonly used chemical activation procedures. Lastly, we compared the effect of the activation protocol on embryo ploidy.

## Results

### Validation of the intracytoplasmic injection technique for bovine oocytes

**Figure 1:**
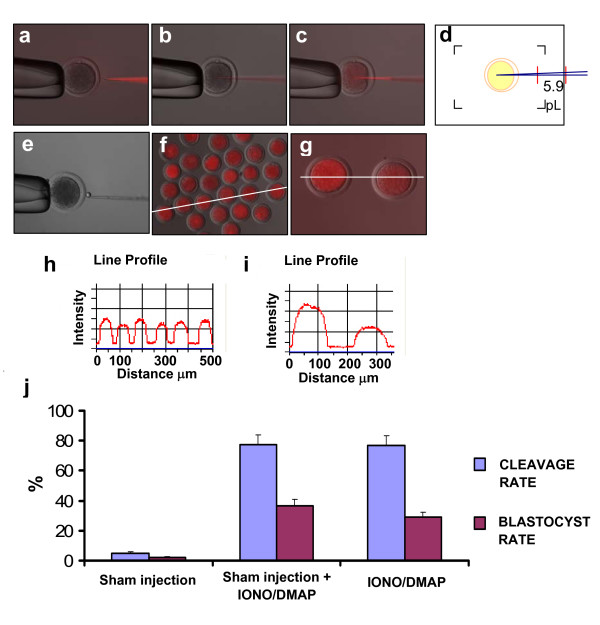
**Validation of intracytoplasmic injection technique**. a, b, c: Sequence of injection. a) Pipette loaded with Texas Red dextran just before injection. b) Pipette advanced into the oocyte; cytoplasm is aspirated to break the plasma membrane. c) Aspirated cytoplasm and Texas Red dextran are injected into the oocyte. d) Schematic representation of the microscope reticulum used as guide to control the injected volume. The oocyte is represented in yellow and the pipette in blue. The red lines indicate the volume introduced into the oocyte which, calculated measuring the pipette internal diameters at both ends, is 5.9 pL. e) An oil drop of the same size as the injected volume is shown next to an oocyte. f) Oocytes injected using Texas Red dextran. g) From left to right, oocyte injected 2X and 1X the normal volume of Texas Red dextran. h) Fluorescent intensity profile of the line shown in f. i) Fluorescent intensity profile of the line shown in g. j) Developmental rates of injected and uninjected bovine oocytes after activation using ionomycin/DMAP.

Intracytoplasmic injections into bovine oocytes represent a challenge because of the high elasticity of the plasma membrane and the opacity and darkness of these oocytes. In this study, we adapted the technique used for ICSI to inject consistent volumes of PLCZ1 cRNA into bovine MII oocytes. Using this technique, we were able to inject a precise amount of media, confident of having penetrated the plasma membrane. To achieve this, a determined volume of media was loaded into a Fluorinert-filled pipette using a hydraulic microinjector. Then, the pipette was advanced into the oocyte up to about three-quarters of its diameter. By applying negative pressure, the oocyte cytoplasm was slowly aspirated. A well-defined meniscus was observed at the interface of the oocyte cytoplasm and the media when the plasma membrane was intact. When the plasma membrane was broken, the meniscus disappeared, and the flow of cytoplasm into the pipette was faster as a consequence of lower resistance. These indicators were used to determine that the membrane had been penetrated. Then, applying positive pressure, the cytoplasm was injected back into the oocyte, followed by the media containing cRNA (Figure 1a–c). The volume of media injected was controlled by observing the meniscus at the interface of media and Fluorinert, guided by the reticulum present in the microscope's field of view (Figure 1d). According to our measurements of the internal diameter of the pipette and the length of the injected column of media, we calculated that the injection volume would be ~6 pL.

Measuring the diameter of oil drops released in aqueous media, we calculated an injection volume of 7 ± 0.2 pL (range 6 to 8.2 pL) (Figure 1e).

To corroborate the efficiency of the injection technique, we injected Texas Red dextran into the oocytes and then checked, under fluorescence excitation, the number of oocytes that had retained the dye. Out of 101 attempted injections, 99 resulted in successful injection of oocytes with clear red fluorescence in their cytoplasm (Figure 1f). Subsequent activation of these oocytes – using ionomycin/DMAP – induced parthenogenetic development at similar rates to noninjected controls (data not shown). Moreover, the fluorescence intensity observed in the oocytes was similar among injected oocytes, indicating that the volume of media injected was consistent from oocyte to oocyte (Figure 1f–i). Finally, parthenogenetically-activated (Ionomycin/DMAP) sham injected oocytes developed at similar rates than noninjected controls (Figure 1j).

### Activation and parthenogenetic development of bovine oocytes injected with PLCZ1 cRNA

We have previously shown that injection of mPLCZ1 (mouse PLCZ1) cRNA into bovine oocytes induces long-lasting [Ca^2+^]_i _oscillations [23]. However, in those studies, we did not investigate the ability of mPLCZ1 to induce oocyte activation or parthenogenetic development. In addition, whether or not injection of bPLCZ1 (bovine PLCZ) cRNA could replicate the responses induced by bull sperm was not ascertained. To answer these pending questions, we first determined whether mPLCZ1 cRNA was able to induce oocyte activation, which was monitored by the extrusion of the second polar body. When bovine oocytes were injected 22 hours after the onset of maturation, extrusion of the second polar body was observed in all oocytes (n = 13) within five hours of PLCZ1 cRNA injection. More importantly, rates of oocyte cleavage to the two-cell stage and pre-implantation embryo development to the blastocyst stage were comparable to those observed for oocytes activated by Ionomycin/DMAP (Table 1). We next examined whether injection of bPLCZ1 cRNA was able to induce activation and embryo development in these oocytes. As shown in Table 2 (tenth dilutions), bPLCZ1 effectively induced activation and embryo development to the blastocyst stage. We then investigated whether an association could be established between cRNA concentrations and high rates of pre-implantation embryo development. We first examined tenth dilutions of our cRNA stock, and then refined the concentrations to obtain maximum embryo development. Among the concentrations tested, mPLCZ1 cRNA was most effective when used at concentrations ranging from 0.25 to 1 μg/μL (Table 3), whereas bPLCZ1 cRNA was effective at concentrations nearly 5-fold lower (Tables 2 and 3). Importantly, unlike mPLCZ1, the highest concentrations of bPLCZ1 cRNA tested here had negative effects both on cleavage and blastocyst rates (Table 2).

**Table 1:** Parthenogenetic embryo development induced by injection of mouse PLCZ1 cRNA into bovine oocytes (3 replicates).

	Ionomycin/DMAP	mPLCZ1
Oocytes (n)	161	123
Cleavage rate^1^	81	88
Blastocyst rate^1^	18	23
Blastocyst cell number^2^	63 ± 16	75 ± 17

^1 ^Percentage from activated oocytes

^2 ^Mean ± standard error

**Table 2:** Optimization of bovine PLCZ1 cRNA concentration to activate bovine oocytes.

Experiment	bPLCZ1 cRNA concentration	Replicates	Oocytes injected	Cleavage Rate^1^	Blastocyst Rate^1^
Tenth dilutions	1 μg/μL	3	110	49.1^a^	12.7^a^
	0.1 μg/μL	3	118	87.3^b^	29.7^b^
	0.01 μg/μL	3	112	69.6^a^	15.2^a^

Refined dilutions	0.5 μg/μL	4	144	70.8^a^	18.8^a^
	0.1 μg/μL	4	154	88.3^ab^	29.9^a^
	0.05 μg/μL	4	145	89.0^b^	22.8^a^

^a,b^: Percentages not sharing a common letter within experiment are statistically different (P < 0.05, *Chi-*square test).

^1 ^Percentage from activated oocytes.

**Table 3:** Optimization of mouse PLCZ1 cRNA concentration to activate bovine oocytes.

Experiment	mPLCZ1 cRNA concentration	Replicates	Oocytes injected	Cleavage Rate^1^	Blastocyst Rate^1^
Tenth dilutions	0.5 μg/μL	3	123	90.2^a^	32.5^a^
	0.05 μg/μL	3	112	83.9^b^	8.9^b^
	0.005 μg/μL	3	122	48.4^c^	0.8^c^

Refined dilutions	1 μg/μL	3	107	79.4^a^	33.6^a^
	0.5 μg/μL	3	112	92.0^a^	33.9^a^
	0.25 μg/μL	3	110	85.5^a^	33.6^a^

^a,b,c^: Percentages not sharing a common letter within experiment are statistically different (P < 0.05, *Chi-*square test).

^1 ^Percentage from activated oocytes

**Figure 2:**
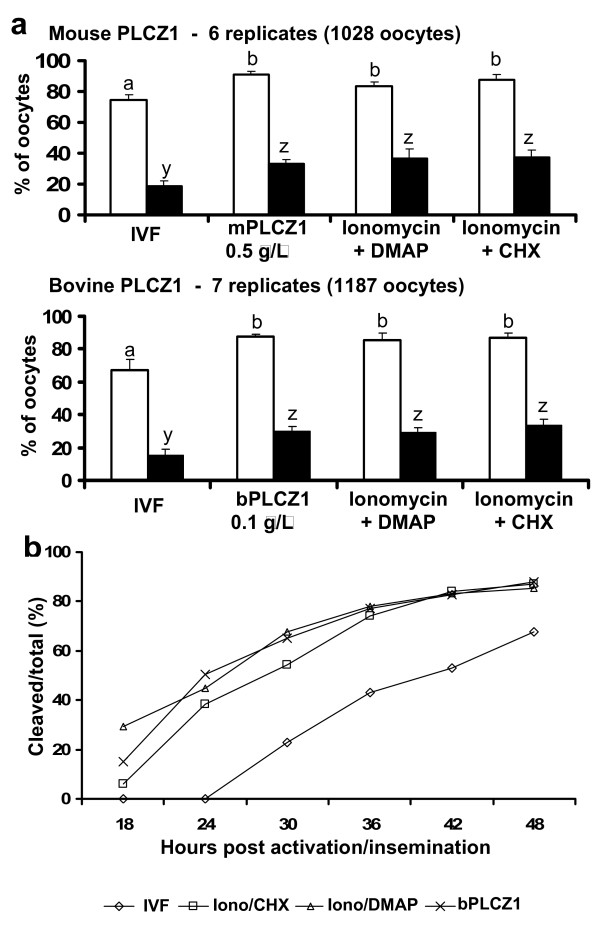
**PLCZ1 cRNA injection induces bovine parthenogenetic development at similar rates to common chemical activation protocols**. a) Mean cleavage (open bars) and blastocyst (black bars) rates after IVF or parthenogenetic activation using different methods. Error bar represents SEM. a, b or y, z: different superscripts represent P < 0.05. b) Cleavage of embryos after IVF or parthenogenetic activation using different methods. Iono = ionomycin; CHX = cycloheximide.

With the optimal concentrations of m and bPLCZ1 cRNAs determined, we investigated whether PLCZ1 cRNAs induced pre-implantation embryo development to the blastocyst stage at rates comparable to those induced by IVF and by frequently used parthenogenetic procedures. Cleavage and blastocyst rates were similar among parthenogenetic embryos regardless of the activation method (Figure 2a), and were higher than those achieved by IVF-derived embryos (P < 0.05). Also, parthenogenetically activated zygotes consistently cleaved to the two-cell stage at earlier times than IVF embryos (Figure 2b). Among parthenotes, a higher proportion of DMAP-activated embryos had cleaved by 18 hours postactivation, but no differences were observed thereafter (Figure 2b). Collectively, our results show that injection of PLCZ1 cRNAs induces high rates of pre-implantation bovine embryo development.

### Injection of PLCZ1 cRNAs induces [Ca^2+^]_i _oscillations and IP3R-1 down regulation in bovine oocytes

**Figure 3:**
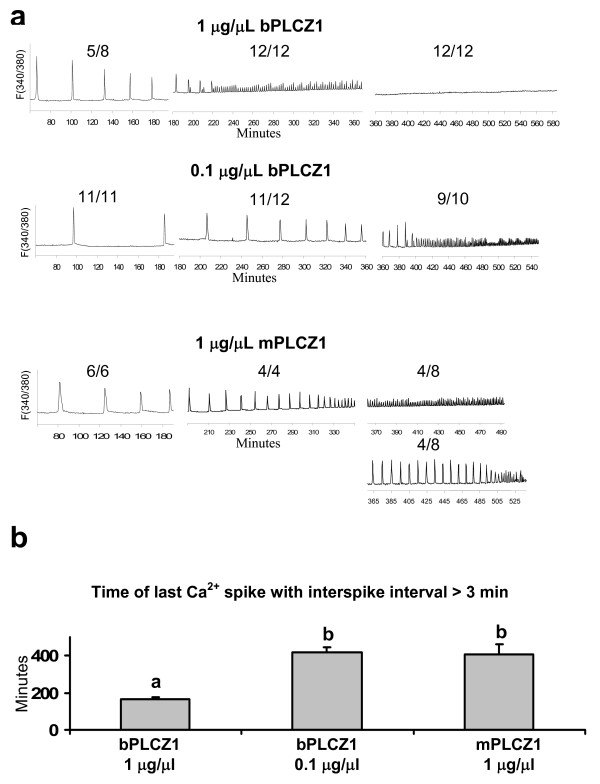
**PLCZ1 cRNA injection induces sperm-like calcium oscillations**. a) Representative [Ca^2+^]_i _profiles. The fluorescence intensity ratio at 340/380 nm is plotted over time (minutes after PLCZ1 injection). The number above each graph represents the proportion of oocytes analyzed that displayed a similar pattern to that shown. b) Minutes after injection at which the [Ca^2+^]_i _pattern changed from interspike intervals of > 3 minutes to < 3 minutes for each treatment. Data represented as mean ± SEM. Different letters indicate P < 0.05.

Our previous finding that concentration and species-of-origin affect PLCZ1 cRNA's ability to induce embryo development suggests that the cRNA concentrations employed in this study might have produced specific [Ca^2+^]_i _responses, as it is well established that too low or excessive [Ca^2+^]_i _stimulation negatively impacts embryo development [1,24]. We therefore investigated the pattern of [Ca^2+^]_i _oscillations induced by PLCZ1 cRNA injected at different concentrations. Our results using mPLCZ1 extend our previous findings [23], and show that sperm-like [Ca^2+^]_i _oscillations are observed only during the first few hours gradually giving way to high frequency [Ca^2+^]_i _oscillations, which likely reflects excessive protein accumulation (Figure 3a). Regarding bPLCZ1, the lowest cRNA concentration tested (0.1 μg/μL), which promoted high rates of embryo development, induced oscillations similar to those triggered by 1 μg/μL mPLCZ1 and, accordingly, high frequency [Ca^2+^]_i _oscillations (less than three minute intervals) were observed by 5 to 6 hours post-cRNA injection (Figure 3b). Injection of 1 μg/μL bPLCZ1 cRNA induced [Ca^2+^]_i _oscillations that transitioned into high frequency oscillations by ~3 hours, and in all evaluated oocytes oscillations had ceased 6 hours after cRNA injection (Figure 3); this cessation of [Ca^2+^]_i _oscillations was not observed either with 0.1 μg/μL bPLCZ1 or with 1 μg/μL mPLCZ1 cRNA.

**Figure 4:**
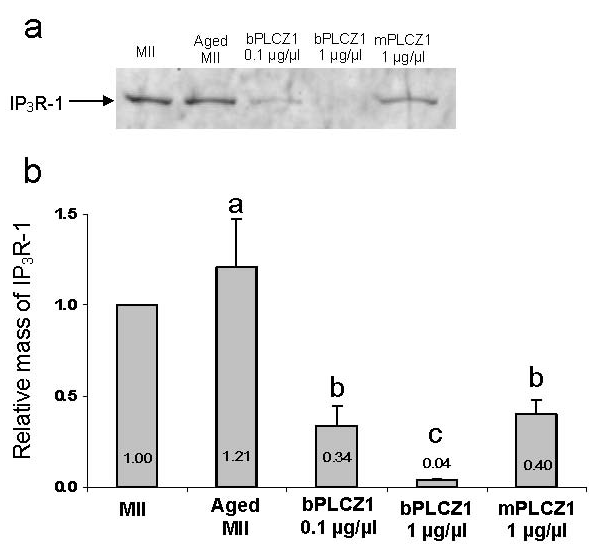
**PLCZ1 cRNA injection induces IP_3 _R-1 downregulation**. a) Immunoblot. Five bovine oocytes were used per lane; samples were collected 12 hours after cRNA injection. MII oocytes were collected at the time of cRNA injection. Aged MII are noninjected oocytes that were left in culture the same amount of time as the injected ones. bPLCZ1 = bovine PLCZ1; mPLCZ1 = mouse PLCZ1. b) Quantification of relative abundance of IP_3_R-1 versus the levels observed in MII oocytes. The number in the bars indicates relative IP_3_R-1 mass. Data represented as mean ± SEM of two replications. Different letters indicate P < 0.06.

Fertilization-associated [Ca^2+^]_i _oscillations are thought to be underlined by steady production of IP_3 _[15] that leads to IP_3_R-1 down-regulation [17]. Accordingly, we evaluated whether injection of PLCZ1 cRNA resulted in IP_3_R-1 degradation, and whether IP_3_R-1 down regulation was associated with PLCZ1 cRNA concentration and species-of-origin. We found that injections of 0.1 μg/μL bPLCZ1 or 1 μg/μL mPLCZ1 cRNA induced fertilization-like IP_3_R-1 degradation (Figure 4), whereas injection of 1 μg/μL bPLCZ1 cRNA depleted IP_3_R-1 from oocytes (Figure 4),

### PLCZ1 cRNA-activated bovine embryos exhibit high degree of normal chromosomal composition

**Figure 5:**
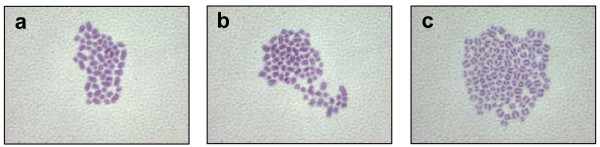
**Representative chromosomal spreads from 8-cell stage bovine embryos (1000×)**. a) Diploid cell, b) triploid cell, and c) tetraploid cell.

Activation of development in oocytes of large domestic species in the absence of fertilization requires the successive application of a Ca^2+ ^ionophore followed by incubation for a few hours with a protein kinase or protein synthesis inhibitor [21]. While these treatments are highly effective at inducing pre-implantation embryo development, they cause high rates of chromosomal abnormalities [25-27]. Given that we have shown that injection of PLCZ1 cRNAs dose-dependently induce high rates of parthenogenetic pre-implantation development, we asked whether a higher proportion of these embryos showed normal chromosomal composition. Accordingly, we compared the chromosomal composition of eight-cell parthenogenetic embryos generated by injection of PLCZ1 cRNAs, versus that of embryos activated by two common chemical activation procedures, as well as of IVF-derived embryos (Table 4; Figure 5). Embryos generated using ionomycin/DMAP showed the highest proportion of abnormal ploidy (70%), while embryos activated using ionomycin/cycloheximide showed a modest amount of aneuploidy (33%) and IVF-derived embryos the least (6%). PLCZ1 cRNA-activated embryos exhibited the lowest percentage of aneuploid embryos (25%) among the parthenogenetic treatments, although it was not significantly different than CHX-activated embryos. These results demonstrate that injection of PLCZ1 cRNA is effective at inducing development of parthenogenetic embryos with high rates of normal chromosomal composition.

**Table 4:** Chromosomal composition of parthenogenetic 8-cell embryos activated using different protocols.

	Embryos Evaluated	Informative embryos	2n	3n	4n	Mixoploid (2n/4n)	Other	Total Abnormal
IVF	22	17	16 (94%)	0	1 (6%)	0	0	1 (6%)^a^
mPLCZ1	28	16	12 (75%)	0	4 (25%)	0	0	4 (25%)^ab^
Iono/DMAP	26	20	6 (30%)	0	10 (50%)	2 (10%)	2 (10%)	14 (70%)^c^
Iono/CHX	28	18	12 (67%)	1 (5%)	5 (26%)	0	1	6 (33%)^b^
Total	104	71						

^a,b,c^: Percentages not sharing a common letter within experiment are statistically different (P < 0.05, *Chi*-square test).

A total of 167 metaphases were evaluated (2.3 per informative embryo).

## Discussion

A growing body of evidence supports the view that oocyte activation during mammalian fertilization is triggered by the delivery of the sperm-specific PLCZ1 [7]. We show that expression of both m and bPLCZ1 in bovine oocytes induces high rates of oocyte activation and pre-implantation embryo development in vitro by initiating fertilization-like [Ca^2+^]_i _oscillations in these oocytes. We also found that lower concentrations of bPLCZ1 than mPLCZ1 cRNA were required to induce [Ca^2+^]_i _oscillations and high rates of parthenogenetic development. Lastly, given the high proportion of normal chromosomal composition among embryos activated by injection of PLCZ1 cRNA, this procedure might be more desirable for inducing oocyte activation in assisted reproductive techniques such as SCNT and ICSI.

### PLCZ1 and parthenogenetic development

Our results show that a single injection of m or bPLCZ1 cRNA into bovine oocytes induces rates of oocyte activation and pre-implantation parthenogenetic development that are comparable to those observed after chemical activation. Unlike the most commonly used chemical procedures, where the [Ca^2+^]_i _rise induced by ionomycin is monotonic, and unable to persistently subdue MPF activity [28], requiring the use of protein synthesis or protein kinase inhibitors to do so, injection of PLCZ1 cRNA alone is able to induce all events of oocyte activation. Nonetheless, the effectiveness of PLCZ1 cRNA was dose-dependent, with either too low or too high of concentrations having detrimental effects on embryo development. Thus, while PLCZ1 cRNA injection may require dose optimization for the species of interest, it may serve as an advantageous alternative to chemical activation protocols to induce parthenogenetic and SCNT embryo development. Because the normal fertilization [Ca^2+^]_i _pattern can be closely replicated by injection of PLCZ1 cRNA, it would be possible to discern whether or not reprogramming defects commonly associated with nuclear transfer-cloning are related to aberrant [Ca^2+^]_i _signaling. In addition, injection of PLCZ1 cRNA may also be useful to stimulate oocyte activation and embryo development following bovine ICSI, given that this procedure is highly inefficient in this species with the inability of injected sperm to initiate [Ca^2+^]_i _oscillations as one of the underlying problems [29].

In the present study, cleavage rates and embryo development to the blastocyst stage were higher in parthenotes than in IVF-derived embryos. We speculate that because all oocytes used for parthenogenetic activation were denuded prior to activation, which allowed for selection based on the presence of a polar body and evenly granulated cytoplasm, a higher proportion of developmentally competent oocytes might have been selected for these procedures. Conversely, IVF was performed in cumulus-enclosed oocytes, which might have resulted in fertilization of some immature or abnormal oocytes, which inherently have lower developmental competence. Parthenogenetically activated embryos also cleaved earlier than IVF-derived embryos. We hypothesize that while in the case of parthenotes the time of activation is synchronized by ionomycin treatment or injection of PLCZ1 cRNA, sperm entry during IVF takes place within a period of 6 hours [30]. Interestingly, within the parthenogenetic procedures, embryos activated with ionomycin/DMAP cleaved earlier than those activated by ionomycin/cycloheximide. The basis for this difference remains unclear, although a more rapid decline in MPF and MAPK is reportedly associated with the use of DMAP [31].

Parthenogenetic procedures commonly used for bovine oocytes are associated with high rates of abnormal chromosome composition [25-27]. Moreover, the frequency of aneuploid embryos was greater when DMAP was used in the activation protocol [25]; our study showed analogous results. Importantly, injection of PLCZ1 cRNA effectively activated bovine oocytes without causing significant increase in the frequency of aneuploid embryos when compared to IVF-derived embryos, as only a quarter of PLCZ1-activated embryos were tetraploid, which is within the range of chromosomal abnormalities reportedly found in bovine IVF embryos (15 to 30 percent) [32,33]. It is worth noting that some tetraploid embryos may arise, at least in the case of PLCZ1 cRNA injection, as a result of the use of cytochalasin B, which is required to obtain diploidization of parthenogenetic zygotes; therefore, this complication is unlikely to be of concern if PLCZ1 cRNA were to be used in ICSI procedures, which will not require the use of this drug.

### PLCZ1 and [Ca^2+^]_i _oscillations in bovine oocytes

Here we show that injection of PLCZ1 cRNAs into bovine oocytes induced long-lasting [Ca^2+^]_i _oscillations that were similar to those induced by the sperm in this species. The injection of the PLCZ1 cRNAs was directly responsible for the [Ca^2+^]_i _responses, as [Ca^2+^]_i _oscillations were precluded when PLCZ1 cRNA injection took place in the presence of cycloheximide (data not shown), which inhibited cRNA translation. Likewise, [Ca^2+^]_i _oscillations were not observed when matured oocytes were injected with PLCδ1 cRNA, a closely related family member (data not shown).

In bovine oocytes, fertilization-associated [Ca^2+^]_i _oscillations occur approximately every 20 minutes [34], although variable [Ca^2+^]_i _oscillations patterns were observed, with one to five [Ca^2+^]_i _transients recorded during a 60-minute period [35], possibly due to highly variable oocyte quality. Injection of PLCZ1 cRNA into bovine oocytes nearly replicated the sperm-induced [Ca^2+^]_i _oscillatory pattern, at least for the first few hours after injection. Inevitably, and regardless of concentration and species of origin, the frequency of PLCZ1-induced oscillations increased, with oscillations occurring less than three minutes apart. This acceleration of oscillations likely reflects protein accumulation as a result of persistent translation of the cRNAs and it was most evident after the injection of high concentrations of bPLCZ1 cRNA. What it is more, in the latter case, oscillations ceased within six hours after the injection. The underlying mechanism responsible for the termination of the oscillations is not yet clear, although the major downregulation of IP_3_R-1 observed in these oocytes might be a contributing factor [17]. Other factors, such as IP_3_R-1 dephosphorylation [18,36], or endoplasmic reticulum reorganization [37,38] cannot be discounted.

The cRNA concentrations that stimulated too low or too high [Ca^2+^]_i _responses produced the lowest cleavage and developmental rates. These data are consistent with previous reports in the literature showing the regulatory role of [Ca^2+^]_i _oscillations on the initiation and completion of oocyte activation events [1,39,40]. For example, it was shown that fewer number of [Ca^2+^]_i _transients are required to initiate events of oocyte activation than what is required to complete these events [1]. It is therefore reasonable to speculate that the shorter duration of the [Ca^2+^]_i _transients observed after injection of 1 μg/μL bPLCZ1 might have been insufficient to complete all the oocyte activation events. Alternatively, the overwhelming stimulation of the phosphoinositide pathway under these conditions might have also negatively impacted cellular functions, thereby compromising embryo development. Importantly, our observation that excessive [Ca^2+^]_i _oscillations induced by injection of bPLCZ1 cRNA result in lower cleavage rates and embryo development is consistent with previous reports indicating that injection of high concentrations of PLCZ1 cRNAs into mouse oocytes had detrimental effects on embryo development [10,41].

The observation that injection of PLCZ1 cRNAs efficiently induces IP_3_R-1 degradation in bovine oocytes is consistent with the notion that PLCZ1-initiated [Ca^2+^]_i _oscillations involve the phosphoinositide pathway and IP_3 _production [42,43], as IP_3_R-1 degradation requires IP_3 _binding to its receptor, followed by ubiquitination and proteasomal degradation of the receptor [44]. It is worth noting that neither Ca^2+ ^release nor cell cycle progression induce IP_3_R-1 degradation in mammalian oocytes [16,17].

Injection of sperm preparations from a variety of mammalian species triggered fertilization-like [Ca^2+^]_i _oscillations in oocytes from different species, suggesting that the sperm factor is not species-specific [45-47]. Consistent with this view, injection of PLCZ1 cRNA coding for the human, simian, and mouse proteins induced [Ca^2+^]_i _oscillations and parthenogenetic development of oocytes from non-homologous species [41]. Here we extend those results and show that mPLCZ1 induces [Ca^2+^]_i _oscillations and parthenogenetic development in bovine oocytes. Lower bPLCZ1 cRNA concentrations were required to induce [Ca^2+^]_i _oscillations and activation responses comparable to those induced by mPLCZ1 cRNA. Moreover, the opposite response was observed when m and b PLCZ1 cRNAs were injected in mouse oocytes (data not shown). There are several possibilities to explain the apparent higher specific activity of bPLCZ1 cRNA in our studies. The first possibility is that bPLCZ1 protein exhibits higher specific activity than mPLCZ1 in bovine oocytes, and this might be due to higher affinity for the endogenous substrate, or alternatively, because the distribution of bPLCZ1 protein more closely overlaps with that of its substrate. A second possibility that cannot be discounted is that species-specific differences in cRNA translation efficiency result in higher concentrations of bPLCZ1 protein than mPLCZ1 protein. Although this is possible, we considered this possibility improbable as the used cRNAs coded only for the open reading frame, were produced under identical conditions and used the same backbone vector. Regardless, additional in vivo and in vitro studies will be required to determine which of these possibilities is correct.

A previous study also compared the oscillatory activity of different PLCZ1s, but in mouse oocytes [41]. It was shown that human PLCZ1 was more effective at inducing [Ca^2+^]_i _oscillations in mouse oocytes than simian or mouse PLCZ1 [41]. Nevertheless, our data represents the first indication that some PLCZ1 may be more effective within species than across species. It will be interesting to determine if gene sequence differences among PLCZ1s from different species, which are mainly in the inter-domain regions, the linker region, may play any role in conferring species-specific adaptations. Therefore, while the mechanism of initiation of [Ca^2+^]_i _oscillations in mammals is highly conserved, adaptations may have occurred during evolution to carefully orchestrate the early events of oocyte activation, the appropriate conclusion of which will impact embryo development to term.

## Conclusion

We show that injection of m and bPLCZ1 cRNA into bovine oocytes induces dose-dependent [Ca^2+^]_i _oscillations, IP_3_R-1 downregulation and parthenogenetic development up to the blastocyst stage. In addition, PLCZ1-cRNA-activated embryos displayed low levels of aneuploidy. Hence, PLCZ1 cRNA injection represents an effective tool to activate bovine oocytes and to study the impact of [Ca^2+^]_i _oscillations on mammalian development.

## Methods

All chemicals were purchased from Sigma (St. Louis, MO) unless otherwise specified.

### Oocyte collection and maturation

Bovine ovaries were obtained from a slaughterhouse and transported in physiological saline solution in an insulated container. Upon arrival at the laboratory, the ovaries were rinsed first with warm tap water and then with physiological saline solution. Antral follicles (2 to 8 mm in diameter) were aspirated using an 18-gauge needle into a 50 mL conical tube by applying 60 mm Hg of negative pressure using a vacuum pump (Cook, Australia). Cumulus oocyte complexes (COCs), with evenly granulated oocyte cytoplasm surrounded by more than four compact layers of cumulus cells, were selected and washed three times in HEPES-buffered HECM medium [48] (HH; 114 mM NaCl, 3.2 mM KCl, 2 mM CaCl2, 0.5 mM MgCl2, 0.1 mM Na pyruvate, 2 mM NaHCO3, 10 mM HEPES, 17 mM Na lactate, 1X MEM nonessential amino acids, 100 IU/mL penicillin G, 100 μg/mL streptomycin, 3 mg/mL BSA). COCs were then matured in Medium 199 supplemented with 10 percent FBS (HyClone, Logan, UT), 1 μg/mL of FSH (Sioux Biochem, Sioux City, IA), 1 μg/mL of LH (Sioux Biochem, Sioux City, IA), 1 μg/mL 17β-estradiol, 2.3 mM of sodium pyruvate, and 25 μg/mL of gentamicin sulphate (Gibco, Grand Island, NY).

### PLCZ1 complementary RNA (cRNA) preparation

A pBluescript vector containing the full-length coding sequence of murine PLCZ1 or pGEMT-easy vector of bovine PLCZ1 was linearized with EcoR1 for mouse PLCZ1 and Sal I for bovine PLCZ1 and used as template for in vitro transcription by the T7 mMessage mMachine High Yield Capped RNA Transcription Kit (Ambion, Austin, TX), following manufacturer instructions. Then, a poly-A tail was added to the cRNA using the Poly(A) Tailing Kit (Ambion). Finally, the cRNA was purified using the Mega Clear Kit (Ambion) and stored at -80°C in single-use aliquots.

Just before use, the cRNA was thawed on ice, heated to 85°C for three minutes, and centrifuged at 13,000 RPM at 4°C for five minutes. Then appropriate dilutions in RNAse-/DNAse-free water (Ambion) were prepared.

### cRNA microinjection

For cRNA injection, a Petri dish containing a 1 μL drop of cRNA and a 50 μL drop of HH under mineral oil was placed on an inverted microscope (TE2000-U, Nikon, Japan) equipped with micromanipulation equipment (Narishige, Japan) at room temperature. After removing the cumulus cells, MII oocytes were placed in the HH media and injected using a beveled micropipette (5 μm internal diameter, MIC-50-0, Humagen, Charlottesville, VA) loaded with Fluorinert, using hydraulic microinjection equipment (Eppendorf, Westbury, NY). cRNA was loaded from the tip of the pipette each time before microinjection. Then, the pipette was advanced into the oocytes and the cytoplasm was aspirated by applying negative pressure to ensure plasma membrane breakage. Finally, the aspirated cytoplasm, followed by the cRNA, was injected into the oocyte by applying positive pressure. The amount of PLCZ1 cRNA injected was controlled by observing the meniscus at the cRNA-Fluorinert interface.

### Intracellular calcium monitoring

Matured oocytes were injected with 0.5 mM Fura-2 dextran (MW 10,000, Molecular Probes, Eugene, OR) as described for PLCZ1 cRNA. Oocytes were monitored in groups in 100 μL drops of protein-free TL-Hepes medium placed on a Petri dish with a glass bottom and covered with mineral oil. A 75 W Xenon arc lamp provided the excitation light and excitation wavelengths were of 340 and 380 nm. Wavelengths greater than 510 nm were collected through a 20X objective by Photometrics CCD SensSys camera (Roper Scientific; Tucson, AZ). Fluorescent intensity ratios (340/380 nm) were measured every twenty seconds for up to three hours using the software SimplePCI (C-Imaging System, Cramberry Township, PA).

### Western blot of IP_3_R-1

To assess the down-regulation of IP_3_R-1 protein, cell lysates from 5 bovine oocytes (without cumulus cells) were mixed with 15 μl of 2X SB [49], as described previously [18], and stored at -80°C. Thawed samples were boiled for 3 min and loaded onto NuPAGE Novex 3–8% Tris-Acetate gels (Invitrogen, Carlsbad, CA). After electrophoresis, proteins were transferred onto nitrocellulose membranes (Micron Separations, Westboro, MA). The membranes were blocked by incubation in PBS containing 0.1% Tween (PBST) supplemented with 5% non-fat dry milk for 1.5 h at room temperature and then incubated overnight with a rabbit polyclonal antibody raised against the C-terminal amino acids 2735–2749 of mouse IP_3_R-1 (Rbt03) [50]. The membranes were subsequently washed in PBST and incubated for 1 h with a goat anti-rabbit secondary antibody conjugated with horseradish peroxidase. Membranes were incubated for 1 minute in cheminoluminescence reagent (NEN Life Science Products, Boston, MA) and developed according to manufacturer's instructions. The intensities of IP_3_R-1 bands were assessed using a Kodak 440 Image Station (Rochester, NY) and plotted using Sigma Plot (Jandel Scientific Software, San Rafael, CA). The intensity of the IP_3_R-1 band from bovine MII eggs was given the value of 1 and IP_3_R-1 abundance in other samples were expressed relative to abundance in MII oocytes.

### In vitro fertilization

COCs matured for 24 hours were co-incubated with sperm (10^6 ^spermatozoa/mL) in a fertilization medium consisting of IVF-TALP (Tyrode's solution) [51] supplemented with 10 mM sodium lactate, 1 mM sodium pyruvate, 6 mg/ml BSA, 50 μg/mL heparin, 40 μM hypotaurine, 80 μM penicillamine, and 10 μM epinephrine) at 38.5°C in 5% CO_2 _in air for 20 hours. Presumptive zygotes were vortexed for two minutes to separate cumulus cells. Groups of 40 to 50 presumptive zygotes were cultured in 400 μL drops of KSOM (Chemicon, Temecula, CA) supplemented with 3 mg/mL BSA under mineral oil at 38.5°C, 5% CO_2 _in air, and humidity to saturation. Seventy-two hours after insemination, 5% FBS was added to the culture media.

### Parthenogenetic activation

Oocytes that had matured for 20 to 22 hours were separated from the surrounding cumulus cells by vortexing in HH medium containing hyaluronidase (1 mg/mL) for 5 minutes. MII oocytes were selected based on the presence of a polar body. Twenty-two to 24 hours postmaturation, oocytes were injected with PLCZ1 and cultured 5 hours in KSOM containing 7.5 μg/mL cytochalasin B to generate diploid embryos. For chemical activation the oocytes were exposed to 5 μM ionomycin (Calbiochem, San Diego, CA) in HH medium for four minutes, then rinsed three times in HH medium and allocated to either four hours culture in 2 mM DMAP in KSOM or six hours culture in 10 μg/mL cycloheximide (CHX) and 5 μg/mL cytochalasin B in KSOM. After these treatments, oocytes were rinsed five times in HH media and cultured as described for IVF embryos.

### Chromosomal Analysis

Seventy-two hours after activation/fertilization, eight-to sixteen-cell embryos were cultured in KSOM-BSA plus 5% FBS containing colcemid for 12 to 14 hours. Then, embryos were exposed to a hypotonic 1% sodium citrate solution for three to five minutes to induce nuclear swelling. Subsequently, embryos were placed on a clean glass slide in a small volume of media. A methanol-acetic acid solution (1:1) was dropped on the embryos while gently blowing with the slides placed under the stereoscope. Then, just before the solution dried, another drop of methanol-acetic acid solution was placed on the embryos and allowed to dry for at least 24 hours at room temperature. After drying, slides were stained with 5% Giemsa solution (Invitrogen, Carlsbad, CA) for ten minutes. Chromosome spreads were evaluated at ×1000 magnification with oil immersion optics (Nikon, Japan). Embryos were classified as being haploid, diploid, triploid, tetraploid, polyploid, and mixoploid.

### Statistical analysis

Cleavage and blastocyst rates were analyzed by chi-square tests when up to 4 replicates were available. When more than 4 replicates were performed, cleavage and blastocyst rate were analyzed using a one-way ANOVA approach in SAS (Carry, NC), with treatment as fixed effect. Similar approach was used also to analyze the continuous variables. Comparisons among treatments were performed using contrast statements. The proportion of embryos with abnormal ploidy was analyzed by a chi-square test.

## Authors' contributions

PJR participated in the study conception and design, carried out the experiments, performed the statistical analysis and data interpretation, and drafted the manuscript. ZB and AI helped with embryo development studies. SYY prepared the cRNAs, carried out the immunoblots, and helped with calcium measurements. CM helped with manuscript writing. KS provided bPLCZ1 clone. RAF and JBC participated in the study conception, design, and manuscript writing. All authors read and approved the final manuscript.
